# Effect of nicotine 6 mg gum on urges to smoke, a randomized clinical trial

**DOI:** 10.1186/s40360-019-0368-9

**Published:** 2019-11-21

**Authors:** Anna Hansson, Thomas Rasmussen, Roland Perfekt, Elin Hall, Holger Kraiczi

**Affiliations:** 1 Global Clinical Pharmacology, McNeil AB, Box 941, SE-251 09 Helsingborg, Sweden; 2 Quantitative Sciences, McNeil AB, Helsingborg, Sweden; 3 Department of Statistics, Lund University School of Economics and Management, Lund, Sweden

**Keywords:** Nicotine replacement therapy, Smoking cessation, Urges to smoke, Craving relief, Clinical trial

## Abstract

**Background:**

Ability to manage urges to smoke is fundamental to maximizing the chances of success in smoking cessation. Previous studies have linked a higher dose of nicotine in nicotine replacement therapy to a higher success rate for smoking cessation. Thus, this study was performed to compare relief of urges to smoke, up until 5 h following treatment with a new 6 mg nicotine gum versus currently marketed 4 mg nicotine gum.

**Methods:**

This was a randomized crossover clinical study. Following 12 h of abstinence from smoking, either one 6 mg or one 4 mg nicotine gum was given to 240 healthy adult smokers. Thereafter, urges to smoke were scored on a 100 mm Visual Analogue Scale repeatedly over 5 h.

**Results:**

The reductions in urges to smoke over the first 1 and 3 h after administration were statistically significantly greater with 6 mg than 4 mg gum, (*p* < 0.005). A 50% reduction in perceived urges to smoke was reached in 9.4 min with 6 mg gum compared to 16.2 min with 4 mg gum (median values). The median duration of a 50% or more reduction in VAS urges to smoke score was 111 min with the 6 mg gum, versus 74 min for the 4 mg gum.

**Conclusion:**

This study provides evidence that the 6 mg nicotine gum provided a greater reduction, faster and longer relief of urges to smoke than the 4 mg nicotine gum.

**Trial registration:**

EudraCT Number: 2010–023268-42. Study was first entered in EudraCT 2011-02-23.

## Background

It has been suggested that cigarette craving is the most sensitive and consistent predictor of smoking behavior and smoking relapse [1, 2]. Cigarette craving has been shown to contribute to and sustain nicotine addiction [3] and is seen by many smokers as a key barrier to successful cessation of smoking [4]. Three studies have investigated the hypothesis that craving is indeed the mechanism through which pharmacological treatment has an effect on smoking cessation and found evidence of partial mediation [5–7]. Therefore, management of cravings is fundamental to maximizing the chances of success in a quit attempt.

Relief of cravings and withdrawal symptoms represents the primary intended use of nicotine replacement therapy (NRT) and relief of these symptoms is also the principal mechanism of action of NRT in the support of smoking cessation [8]. NRT increases the success rate of attempts to quit smoking by approximately 50% versus placebo [9, 10], however, long-term success rates are low [11].

The dose of nicotine appears to be an important factor for the efficacy of NRT in the treatment of tobacco dependence. A study in almost 3600 subjects demonstrated that a 25 mg nicotine patch was superior to both placebo and a 15 mg patch with regards to 12-month smoking cessation rates [12]. In real life, patients may not use enough doses of oral NRT per day to get the maximum benefit [9], and under-dosing has long been discussed as a problem that results in suboptimal treatment effect [13, 14]. A new gum that contains 6 mg nicotine was therefore developed, with the aim of providing an additional dosing option for smokers who are highly dependent (> 20 cigarettes per day), and/or requiring enhanced craving relief. The new gum contains nicotine resinate as the active ingredient, with addition of sodium hydrogen carbonate to facilitate absorption of nicotine through the oral mucosa. A previous study has shown that the 6 mg gum releases approximately 50% more nicotine than the 4 mg gum [15].

The current study was performed to compare 6 mg gum and 4 mg gum in terms of the urges to smoke over time following single-dose administration to abstinent smokers.

Our hypothesis was that the 6 mg nicotine gum would give rise to a greater reduction in VAS urges-to-smoke measurements in the given timeframe compared to the 4 mg reference gum (the underlying null hypothesis was no treatment difference with an alternative hypothesis of a treatment difference in either direction). The primary objective was to compare single-dose administration of 6 mg gum versus 4 mg gum with respect to urges to smoke during the first 1 and 3 h, respectively, after starting to chew gum. Secondary objectives were between-treatment comparisons of urges to smoke scores during 3, 5, and 10 mins, and 2, 4, and 5 h, and to evaluate the tolerability of the study medications.

## Methods

### Study design

**Fig. 1:**
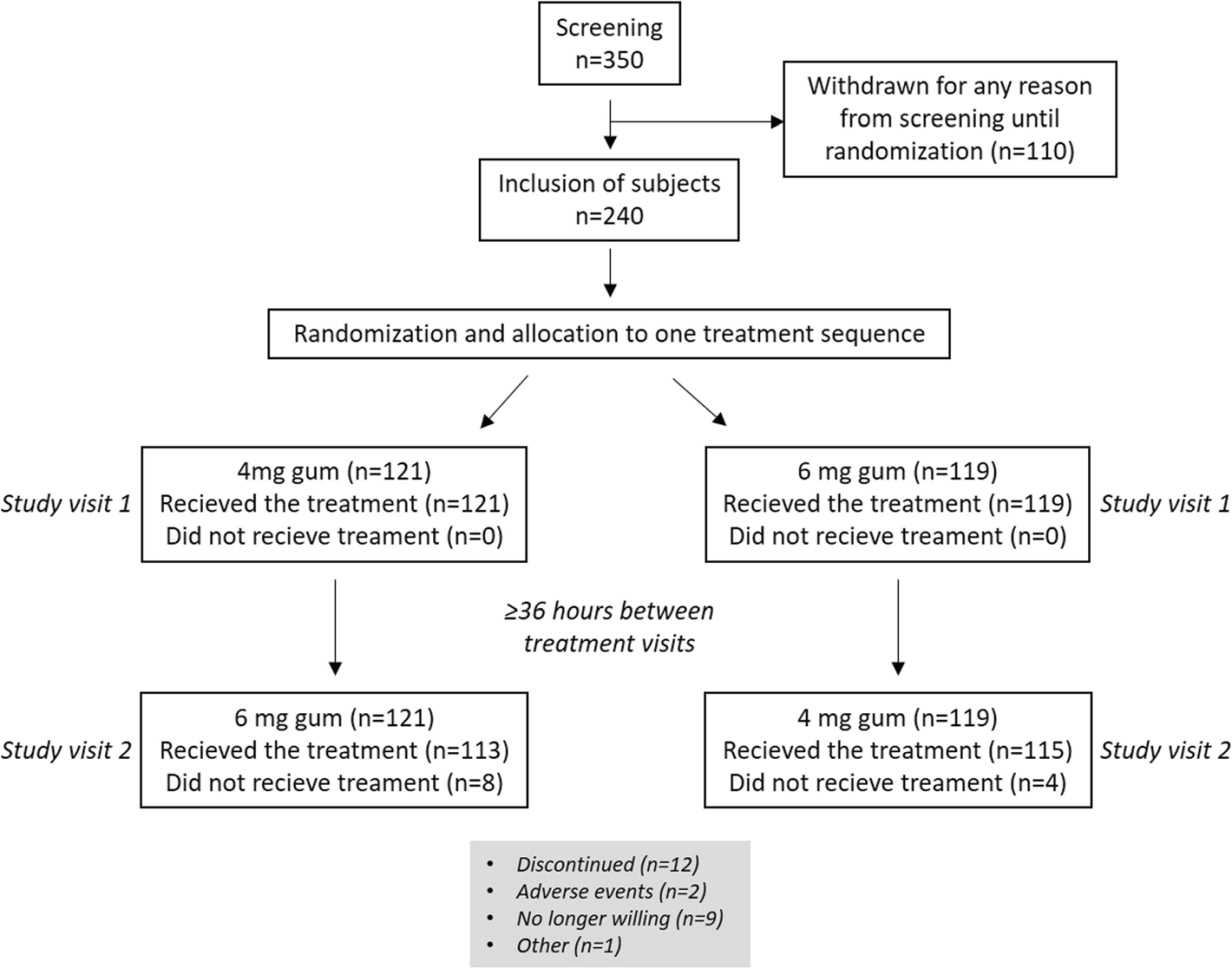
Study design. Flow chart showing the design of the study and number of subjects in each group and study visit as well as number of drop outs

This randomized, 2-way crossover study compared urges-to-smoke in abstinent smokers following single-dose administration of either nicotine 6 mg or 4 mg gum (Fig. 1). Following 12 h of carbon-monoxide verified abstinence, including ≥1.75 h of witnessed abstinence, study treatment was administered and urges to smoke were measured for 5 h. The primary study objective was to compare urges to smoke during the first 1 and 3 h, respectively, after starting to chew the gum. The study protocol was approved by the local Independent Ethics Committee, in Lund, Sweden, and by the Swedish Medical Products Agency. The study was performed in accordance with current International Conference on Harmonization Guidelines on Good Clinical Practice. The study was performed at the Department of Clinical Pharmacology, McNeil AB, Lund, Sweden, and at Karolinska Trial Alliance, Karolinska University Hospital, Huddinge, Stockholm, Sweden, between April and July 2011. The study planned to enrol 250 subjects, and all randomized subjects with at least one valid efficacy endpoint were included in the statistical evaluation.

### Study population

The study recruited, via newspaper and posters, healthy adult volunteer male and female smokers, aged 19–55 years, who had smoked at least 20 cigarettes per day for at least one year directly preceding the study start, but who were willing to abstain from smoking 12 h prior to and during the treatment visits. Eligible subjects had to weigh at least 55 kg and have a body mass index (BMI) of 17.5–32.0 kg/m^2^. Female subjects of childbearing age had to be using an effective form of contraception. Concomitant medication was only allowed if considered necessary for the subject’s welfare and allowed at the discretion of the investigator. A full list of inclusion and exclusion criteria for this study can be found in Additional file 1. All subjects provided written, informed consent before entering the study. Subjects were financially compensated for their participation.

### Study execution

The subjects were randomly allocated in equal proportions to one of two treatment sequences by means of a random number generator. The two study medications comprised nicotine 6 mg gum and nicotine 4 mg gum (Nicorette® Freshfruit 4 mg; McNeil AB, Helsingborg, Sweden) which were administered on two separate visits (Fig. 1). The gums differed in appearance, but gums were concealed from subjects, both prior to and after chewing, and they were not told which gum they received during a specific visit.

In order to conceal the gums from the subjects, gums were transferred from blisters to

labelled, opaque aluminium bags in a separate room. The subjects were to pour the gums straight into their mouths without looking at the contents of the bag. Subjects were then instructed to chew the gums slowly for 30 min, with breaks as they considered most convenient. After chewing, without looking at the gum, each subject was to place the used gum on a piece of foil, which was immediately wrapped around the gum by study personnel. The gums were then placed in the labelled aluminium bag a labelled plastic bag and stored at − 20 °C until transport to the analysis laboratory.

Periods of at least 36 h without NRT separated the two visits. Subjects abstained from smoking or using nicotine-containing products from 12 h before, and throughout, each treatment visit. They were instructed not to smoke after 8 pm on the evening prior to each visit; on arrival at the study site around 7.45 am the following morning, a carbon monoxide monitor was used to verify abstinence from smoking (subjects were rescheduled if CO > 20 ppm). Since it is known that acidic beverages may interfere with buccal absorption of nicotine [16], the study subjects were not allowed to eat or drink at 15 min prior until 60 min after study treatment administration. Administration of study treatment commenced at around 9.30 am. Subjects chewed one piece of gum slowly for 30 min, with breaks in chewing as needed. After chewing, used gums were collected for analysis of residual nicotine. Nicotine was extracted from each gum matrix using a two-phase system. The aqueous phase was analyzed using a High Performance Liquid Chromatography system and nicotine was quantified using a calibration curve. All observed and spontaneously reported adverse events during the study were recorded.

### Measurement and analyses of urges-to-smoke

Subjects were provided with electronic diaries in order to record the time that treatment commenced, and to collect urges-to-smoke data by letting the study subjects answering the question “How strong is your urge to smoke now?”. Urges to smoke were scored on a 100 mm visual analogue scale (VAS) 10 and 3 min before, and at 3, 5, 10, 20, 30, 40, 50 min and 1, 2, 3, 4, 5 h after treatment administration. On the VAS, zero represented ‘no urge to smoke’ and 100 represented ‘extreme urge to smoke’ (Additional file 2). This type of scale is reliable [17, 18] and can be administered quickly and repeatedly, measures the momentary intensity of urges-to-smoke, and are used regularly in smoking research [19–24]. Other cigarette craving scales consist of several individual items. However, a recent study compared the widely used ten-item Questionnaire on Smoking Urges (QSU-brief) with six shorter measures of craving in terms of sensitivity to abstinence and reliability and concluded that the ten-item QSU-brief is not more sensitive to abstinence or reliable than the two-item or a single rating of craving [25]. The reliability and ease of use of 1-item VAS for assessing cravings makes this an appropriate method to have used during this study.

### Study endpoints and statistical analysis

Primary study endpoints were change from baseline in urges-to-smoke. The average score change in urges-to-smoke score in the interval from time zero (baseline) to time *t* was calculated as the difference between the area under the urges-to-smoke- vs.-time curve from time zero to *t* divided by *t*, and the baseline score. Comparisons of the average score change in urges-to-smoke score were based on a mixed linear model that included treatment sequence, treatment, site and period as fixed effects, and subject, nested within sequence, as a random effect. In addition, the baseline urge-to-smoke score was included as a co-varying fixed effect. Pair-wise treatment comparisons of the average score changes from time zero until 3, 5 and 10 min post-administration were performed in the same manner. The sample size calculation was based on a requirement to have at least 90% statistical power for a true mean difference in average score change at 1 h and 3 h of 12.9 mm and 4.3 mm, respectively. The corresponding within subject standard deviations was assumed not to exceed 37.5 mm and 12.5 mm, respectively, based on previous data from a similar crossover study of 6 mg gum vs. 4 mg gum. To adjust for multiplicity a Bonferroni adjustment was made. Consequently, the sample size calculation used a significance level of 2.5%.

In addition to the planned analyses, two post-hoc analyses were performed to investigate speed and duration of relief of urges to smoke. The endpoints for speed of relief were the time points at which the reduction in the VAS urges-to-smoke score first equalled 25, 50 and 75%, respectively, of the baseline value; as estimated using linear interpolation. The quartiles of the distributions of the estimated time to a reduction from baseline by 25, 50 and 75%, with 95% confidence intervals (CI), were estimated separately for each treatment using Kaplan-Meier estimation techniques. Pair-wise treatment comparisons of speed of relief were based on the proportions of subjects with a shorter estimated time-to-event on 6 mg nicotine gum compared to 4 mg gum. To compare treatments with respect to duration of effect, the length of the first periods post-administration during which the VAS score was continuously ≤75%, ≤50% or ≤ 25% of the baseline value were used as endpoints. These were based on follow-up to 5 h post-administration and were calculated using linear interpolation. Treatments were compared using a nonparametric test that involved adjustments for period effects (Mann-Whitney test for period differences between sequence groups, i.e. Koch’s test). For statistical analysis, SAS v 9.2 was used.

## Results

### Descriptive analysis

Two-hundred and forty healthy smokers (120 male, 120 female) were enrolled. The initial plan of enrolling 250 subjects was not met, due to difficulties in recruiting the planned number of subjects in time for study start. Table 1 shows the subject characteristics of the 240 enrolled subjects in the study. A total of 228 subjects completed the study as planned; 12 subjects withdrew prematurely (nine no longer wanted to participate, two because of adverse events, one for other reasons).

**Table 1:** Subject characteristics for enrolled subjects (*n* = 240)

	N	Average (min-max)
Sex	120 male / 120 female	
Ethnicity	235 white, 3 black, 1 Asian	
Age (years)		35 (19–55)
Numbers of cigarettes smoked /day		25 (21–38)
Number of years smoked before study start		18 (2–41)

Mean amounts of 4.0 (±1.28) mg and 2.64 (±0.90) mg nicotine i.e. 67 and 66% of the nicotine content of the gums were released from 6 mg and 4 mg gums, respectively.

**Fig. 2:**
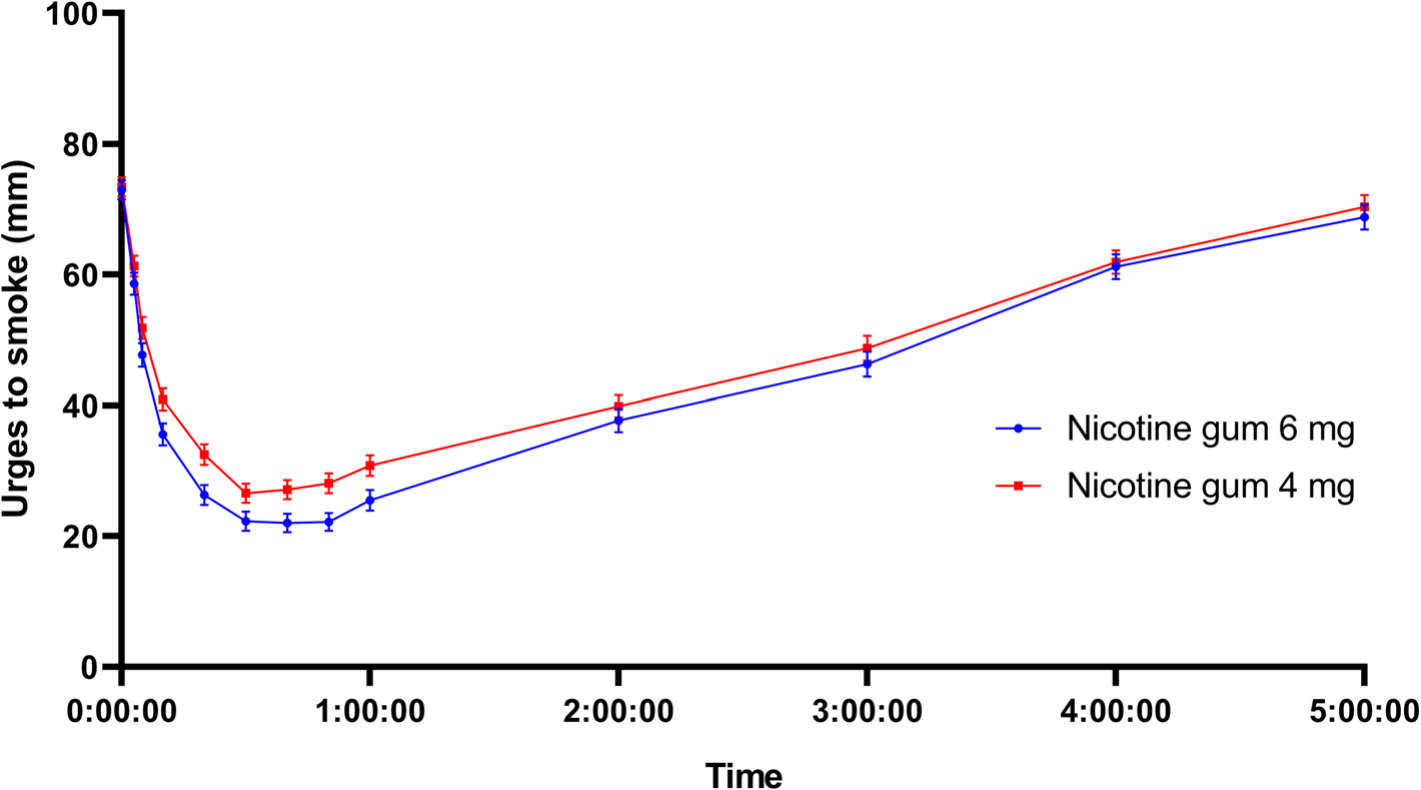
Urges-to-smoke-vs.-time curve. Mean urges-to-smoke-vs.-time curve over 5 h following administration of nicotine 6 mg gum or 4 mg gum to 240 smokers who had abstained from smoking for 12 h

Figure 2 displays the mean urges-to-smoke versus time curves for 6 mg and 4 mg gum over 5 h post-administration. From a mean baseline level of 73 mm (on a 100 mm VAS), 6 mg gum reduced urges to smoke over the first 40 min after treatment start to an average minimum score of 22 mm, i.e. a mean decrease of 51 mm. One hundred and eleven (111) subjects out of 231 (48%) experienced complete or close to complete relief (≤5 mm) during the sampling period. For the 4 mg gum, the average baseline score was 73 mm, the average minimum mean urges-to-smoke score was 26 mm, and 75 subjects out of 234 (32%) had a score of ≤5 mm.

### Primary analysis

Table 2 shows estimated means of subjects’ average changes in urges-to-smoke scores from baseline following administration of 6 mg or 4 mg gum, and the corresponding comparisons between treatments. The 6 mg gum reduced urges to smoke by a mean of 44 mm over the first 60 min and 39 mm over the first 3 h after administration.

**Table 2:** Average change in urges-to-smoke scores between the 6 mg and 4 mg nicotine gum

Time	Average score change from baseline (mm)		
	Gum 6 mg (*n* = 232)	Gum 4 mg (*n* = 236)	6 mg vs 4 mg
3 min	−7.2 ± 9.0	−6.1 ± 8.7	−1.1 [− 2.2, − 0.1], *p* = 0.034
5 min	−12.2 ± 13.4	− 10.4 ± 12.9	−1.9 [− 3.4, − 0.4], *p* = 0.016
10 min	−21.8 ± 18.6	−18.7 ± 17.5	−3.1 [− 5.0, − 1.2], *p* < 0.001
1 h	− 43.9 ± 23.8	−39.3 ± 23.3	−4.8 [− 7.0, − 2.6], p < 0.001^a^
2 h	− 42.7 ± 23.6	− 38.7 ± 23.7	− 4.2 [− 6.2, − 2.1], *p* < 0.01
3 h	− 38.8 ± 23.2	− 35.5 ± 23.4	−3.4 [− 5.8, − 1.0], *p* = 0.004^a^
4 h	− 33.9 ± 22.6	− 31.1 ± 22.7	− 2.9 [− 5.0, − 0.7], *p* = 0.008
5 h	− 28.7 ± 21.8	−26.3 ± 21.5	− 2.5 [− 4.6, − 0.4], *p* = 0.021

^a^Estimate [97.5% CI] and Bonferroni adjusted *p*-value. Estimated mean (±SD) average changes in urges-to-smoke scores over 3, 5 and 10 min and 1, 2, 3, 4 and 5 h following administration of 6 mg gum or 4 mg gum, and corresponding comparisons between treatments (estimated treatment difference [95% CI] and *p*-value)

### Secondary analysis

The mean reductions in urges to smoke were statistically significantly (*p* < 0.05) greater with 6 mg than 4 mg gum for all examined time intervals, including 3, 5 and 10 min.

### Post-hoc analysis

Post-hoc comparisons of the estimated times to 25, 50, and 75% reductions of the baseline urges to smoke scores showed that at all levels of reduction, the times to endpoint were statistically significantly shorter with 6 mg than 4 mg gum.

A 50% reduction in perceived urges to smoke was estimated to be reached in 9.4 min (median with 95% CI, 7.92, 13.4) with 6 mg gum compared to 16.3 min (95% CI 12.07, 19.02) with 4 mg gum (p-value for the null hypothesis of equal time-to-event distributions 0.001).

Similarly, the duration of treatment effect (reduction in urges to smoke) was longer with 6 mg than 4 mg gum; the median duration of a ≥ 50% reduction in urges to smoke was estimated to be 111 min with 6 mg gum versus 74 min with 4 mg gum.

### Safety analysis

All subjects were included in the safety analysis. No serious adverse events were reported. A total of 173 treatment-emergent adverse events were recorded, of which 153 were rated as mild, 18 as moderate and 2 as severe (1 face injury for the 6 mg gum treatment, and 1 cough for the 4 mg treatment). Treatment-emergent adverse events were reported by 68 subjects with 6 mg gum, and 54 subjects with 4 mg gum. The most frequent adverse events were nausea, dyspepsia, throat irritation and hiccups (Table 3). Two subjects withdrew because of adverse events not related to study treatment (one case of cough, and one face injury) but there were no withdrawals due to treatment-related adverse events.

**Table 3:** Treatment-emergent Adverse Events occurring in ≥11 (~ 5%) Subjects in Any Treatment Group

AE Preferred Term	Gum 6 mg (*n* = 232) n (%)	Gum 4 mg (*n* = 236) n (%)
No of Subjects with at least 1 AE	68 (29.3)	54 (22.9)
Dyspepsia	13 (5.6)	6 (2.5)
Nausea	23 (9.9)	15 (6.4)
Throat irritation	22 (9.5)	9 (3.8)

## Discussion

Extensive review of present NRT studies and their data establishes that it is clear that NRT increases the chances of stopping smoking by approximately 50% regardless of NRT type [10]. A previous systemic review of NRT studies also found that there was a benefit to highly dependent smokers when using a 4 mg nicotine gum compared to a 2 mg nicotine gum [11]. At present the strongest forms of oral NRT contain 4 mg of nicotine. A new gum that contains 6 mg nicotine has been developed with the aim of providing enhanced craving relief for highly dependent smokers (> 20 cigarettes per day) who might benefit from a gum with a higher dose of nicotine. A pharmacokinetic study had previously shown that nicotine 6 mg gum attained higher plasma nicotine concentrations at early and late time points post-dose compared to 4 mg gum.

As done in previous pharmacokinetic studies [15], the residual amount of nicotine in used gums was measured. In accordance with the previous results, we found that approximately 50% more nicotine was released from 6 mg gum than 4 mg gum (mean of 4.0 mg vs 2.6 mg nicotine). Conclusions regarding higher plasma levels attained with 6 mg gum in comparison with lower strength gums and 4 mg lozenge drawn in the pharmacokinetic studies are therefore likely to apply also in this study.

Our study enrolled healthy adults who were smoking at least 20 cigarettes per day for at least one year directly preceding the study start, as this is the target population for 6 mg gum in clinical practice. Even though the planned number of study subjects was not fully met, the 240 subjects included, and the 232/236 subjects evaluated was still considered as high enough to be able to reliably evaluate the results of this study, given the 234 evaluable subjects suggested by the sample size calculation. The cross-over design of the study is beneficial since each study subject serve as its own control and it limits the number of needed participants. It does however bring a potential carry-over effect which might influence the results. Urges to smoke was assessed following 12 h of abstinence from smoking, which has been demonstrated to be a reliable method to provoke cravings in a controlled setting [26]. Following single-dose treatment with either 6 mg gum or 4 mg gum, urges to smoke was measured repeatedly over five hours using a 1-item scale. Single rating scales have proven to be as reliable as multi-item scales [25]. Also, VAS has the advantage of using a small scale, which makes it easier to capture small changes between the treatments. However, it also brings the disadvantage of being more difficult to compare between subjects. The within-subject changes in this study do however indicate that this is not a major issue in this study. The crossover design employed in our study permitted within-subject comparisons of the two study treatments. Since the test and reference product differed in appearance, complete blinding of this study was not possible. Hence per strict definition this was an open label trial, with the potential of bias following that type of setting. However, the study subjects were not aware of which treatment they received since the chewing gum was concealed for them both at administration and at removal of the gum. These precautions were taken to eliminate the risk if potential bias from the study subjects.

We found that the 6 mg gum provided statistically significantly greater relief of urges to smoke than 4 mg gum within intervals up to 3, 5, 10 min, 1, 2, 3, 4 and 5 h after start of administration, i.e. 6 mg gums provide a greater craving relief than 4 mg gums in a population of smokers who were highly dependent. The maximum reduction of the average score from baseline was seen in the interval up to 1 h and measured on average 44 mm with 6 mg gum and 39 mm with 4 mg gum and a between treatment difference of 4.8 mm was seen. A difference in urges to smoke score of similar size (3.1 mm) was seen comparing 4 mg nicotine lozenge and 2 mg nicotine lozenge in a previous trial using the same type of scale [19].

As a means to facilitate interpretation of the urges to smoke data and put them into a clinical context, post-hoc analyses were performed. The post-hoc analyses revealed that the higher dose was more effective than the lower dose in terms of speed of onset and duration of treatment effect. The treatment effect within the first 10 min post-administration was greater with 6 mg than 4 mg, and a 50% reduction in perceived urges to smoke was reached in 9.4 min with 6 mg gum compared to 16.2 min with 4 mg gum (median values). Thus, results indicate that 6 mg gum gives a faster craving relief than 4 mg gum. Similarly, the duration of treatment effect appeared to be longer with 6 mg gum; the median duration of a continuous 50% or more reduction in urges to smoke was 111 min with 6 mg gum versus 74 min with 4 mg gum. However, further studies are needed to evaluate potential difference in the long-term smoking cessation success between the 6 mg gum compared to the 4 mg gum.

The adverse events observed with 6 mg gum reflect the events previously reported with lower-strength nicotine gum and included nausea, dyspepsia, throat irritation and hiccups [9, 27–29]. As expected, some adverse events, e.g. dyspepsia, nausea, hiccups and throat irritation were more frequent with 6 mg than 4 mg gum, but none was rated as severe.

## Conclusion

In summary, the 6 mg gum was well tolerated and resulted in a greater reduction in VAS urges to smoke score than 4 mg gum. Greater reductions within the first few minutes post-administration demonstrated faster relief of craving with 6 mg gum, and the duration of effect of 6 mg gum was longer than that of 4 mg gum, indicating that the higher plasma nicotine exposure observed with the 6 mg dose is paralleled by a more pronounced relief of urges to smoke. The efficacy of 4 mg gum in smoking cessation has previously been demonstrated in large, well-designed clinical trials [11, 28–30]. Given the importance of craving management for a successful smoking cessation and the fact that 6 mg gum has proven superior to 4 mg gum in this respect, these properties may prove useful to smokers in a quit attempt.

## Supplementary information

**Additional file 1.** Inclusion and Exclusion criteria. Full list of inclusion and exclusion criteria for the clinical study. Subjects had to meet all of the inclusion criteria and none of the exclusion criteria to be enrolled in the study.

**Additional file 2.** Visual analogue scale for craving measurements. Picture and description of the visual analogue scale used to measure craving.

## Data Availability

The full trial protocol is available upon request from the corresponding author. The datasets used and/or analyzed during the current study are available from the corresponding author on reasonable request.

## Abbreviations

BMI: Body mass index
NRT: Nicotine replacement therapy
VAS: Visual analogue scale
