# Supplementary material for: Effect of nicotine 6 mg gum on urges to smoke, a randomized clinical trial
Source: BMC Pharmacol Toxicol. 2019 Nov 21;20:69. doi: 10.1186/s40360-019-0368-9 (PMC6873734; doi:10.1186/s40360-019-0368-9)
Supplement: Supplementary file 1 — Additional file 1. Inclusion and Exclusion criteria. Full list of inclusion and exclusion criteria for the clinical study. Subjects had to meet all of the inclusion criteria and none of the exclusion criteria to be enrolled in the study. [file 40360_2019_368_MOESM1_ESM.pdf]

Additional file 1.

Inclusion and Exclusion criteria

Inclusion Criteria

Subjects must meet all of the following inclusion criteria to be eligible for enrollment into the study:

1. Healthy male or female subjects between the ages of 19 and 55 years, inclusive. Health is defined as the absence of clinically relevant abnormalities as identified by a detailed medical history, a physical examination, blood pressure, pulse rate and hemoglobin measurements, as judged by the investigator or an authorized physician. The responsible physician may request additional investigations or analyses if necessary.

2. Smoking of more than 20 cigarettes daily during at least one year preceding inclusion.

3. For females: Postmenopausal state (absence of menstrual discharge for at least two years and a serum FSH level exceeding 30 IU/L) or premenopausal/perimenopausal state with an effective means of contraception (oral, injected or implanted hormonal contraceptives, intrauterine device, or status after operative sterilization), or declared absence of sexual contact during the study. Males: No pregnant spouse or partner at screening and willingness to protect their spouse or sexual partners from becoming pregnant during the study.

4. Body Mass Index (BMI) between 17.5 and 32.0 kg/m^2 and a total body weight ≥55.0 kg.

5. A personally signed and dated informed consent document, indicating that the subject has been informed of all pertinent aspects of the study.

6. Willingness and ability to comply with scheduled visits, treatment plan, laboratory tests, and other study procedures specified in the protocol.

Exclusion Criteria

Subjects presenting with any of the following will not be included in the study:

1. Evidence or history of an acute or chronic medical or psychiatric condition or allergy or laboratory abnormality, or of use of drugs that, in the judgment of the investigator or an authorized study physician, increases the risk associated with study participation or interfere with the interpretability of study results.

2. Females: Pregnancy, breast-feeding, premenopausal, or perimenopausal, state with insufficient contraception as specified under Inclusion Criteria. Males with a pregnant spouse or partner or males who are not willing to prevent conception in a spouse or sexual partner.

3. History of regular alcohol consumption in the 6 months before screening, exceeding weekly limits of 2 L of wine or 5 L of beer or 0.6 L of spirits for females, and 3 L of wine or 7.5 L of beer or 0.9 L of spirits for males. The investigator may lower these limits if a subject consumes different types of alcoholic beverages.

4. Treatment with an investigational product, other than those provided in this study between one month preceding the first treatment visit and the last treatment visit of the study.

5. Pathological oral status interfering with normal muscular, sensory, or absorptive function of the oral cavity. Piercing of tongue and lips is considered to impair oral function.

6. Relationship to persons involved directly with the conduct of the study (i.e., principal investigator, subinvestigators, study coordinators, other study personnel, employees or contractors of the sponsor or Johnson & Johnson subsidiaries, and the family of each).
