# Supplementary material for: Effect of nicotine 6 mg gum on urges to smoke, a randomized clinical trial
Source: BMC Pharmacol Toxicol. 2019 Nov 21;20:69. doi: 10.1186/s40360-019-0368-9 (PMC6873734; doi:10.1186/s40360-019-0368-9)

Additional file 2. Visual analogue scale for craving mesurements.

Front of the VAS scale used to measure nicotine craving in the study. Titel of the scale is ”cigarett craving” (Rökbegär), with the scale going from ”No urge to smoke” (inget alls) furthest to the left to ”Extreme urge to smoke” (Värsta tänkbara) furthest to the right.

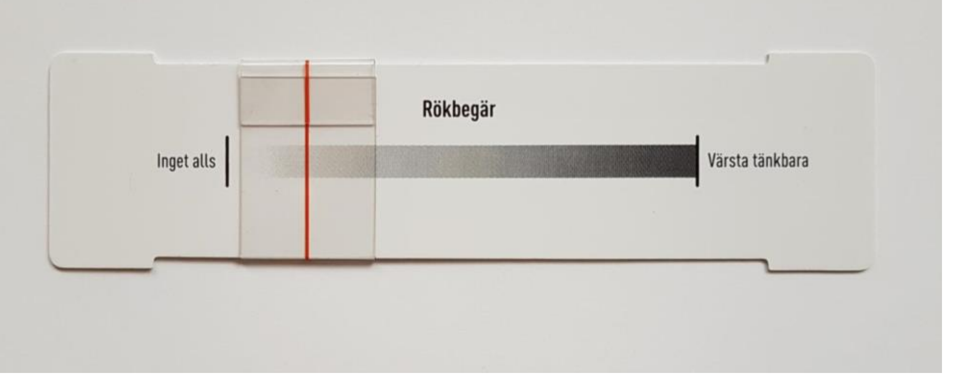

Backside of VAS scale used for measuring nicotine craving.

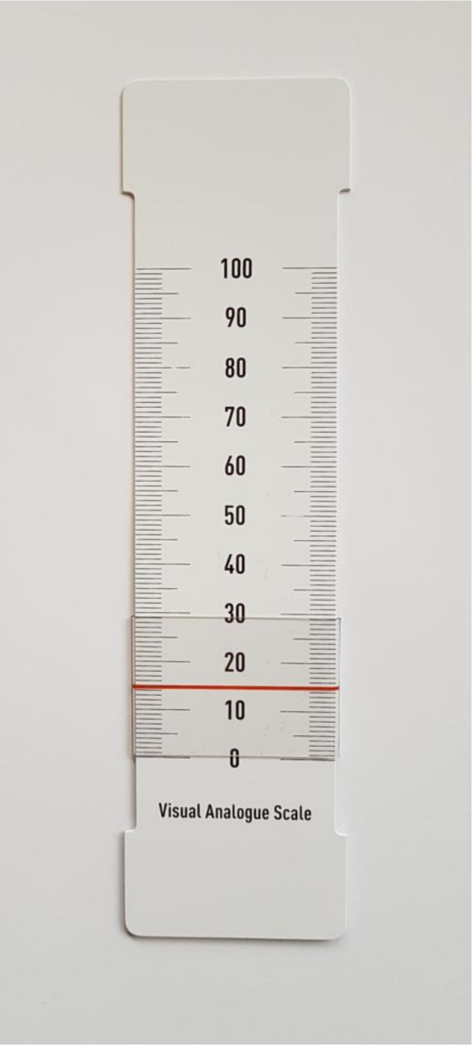

Supplement: Supplementary file 2 — Additional file 2. Visual analogue scale for craving measurements. Picture and description of the visual analogue scale used to measure craving. [file 40360_2019_368_MOESM2_ESM.pdf]
